# The sexual and reproductive health needs of school-going young people in the context of COVID-19 in rural KwaZulu-Natal, South Africa

**DOI:** 10.2989/16085906.2022.2095921

**Published:** 2022-07

**Authors:** Natsayi Chimbindi, Ursula Ngema, Nothando Ngwenya, Andrew Gibbs, Candice Groenewald, Guy Harling, Nondumiso Mthiyane, Busisiwe Nkosi, Janet Seeley, Maryam Shahmanesh

**Affiliations:** 1 Africa Health Research Institute, KwaZulu-Natal, Durban & Mtubatuba, South Africa; 2 Institute for Global Health, University College London, London, United Kingdom; 3 University of KwaZulu-Natal, KwaZulu-Natal, South Africa; 4 Gender and Health Research Unit, South African Medical Research Council, Pretoria, South Africa; 5 Centre for Community Based Research, Human Sciences Research Council, Durban, South Africa; 6 Department of Psychology, Rhodes University, Makhanda, South Africa; 7 MRC/Wits Rural Public Health & Health Transitions Research Unit (Agincourt), University of the Witwatersrand, Johannesburg, South Africa; 8 Department of Epidemiology & Harvard Centre for Population and Development Studies, Harvard TH Chan School of Public Health, Boston, USA; 9 Department of Global Health and Development, London School of Hygiene and Tropical Medicine, London, United Kingdom

**Keywords:** adolescents, pregnancy, HIV, alcohol and substance abuse, COVID-19, Life Orientation

## Abstract

**Background:**

The impact of school closures due to COVID-19 raised widespread concerns about children’s health and well-being. We examine the impact on the sexual health needs of learners in the context of COVID-19 related lockdowns in rural KwaZulu-Natal, South Africa.

**Methods:**

In July–November 2020 and August–November 2021 we conducted 24 in-depth interviews and 8 group discussions with teachers and learners from 4 schools, community members and key education stakeholders. All interviews were conducted by telephone. We used a thematic analysis approach and Nvivo 12 software to manage the data.

**Results:**

Four main themes related to the COVID-19 pandemic emerged from the data: the sexual and reproductive health (SRH) of learners in the lead-up to the pandemic; the impact of COVID-19 on learners’ SRH and wellbeing; the opportunities schools provided to support sexual well-being of learners during the pandemic; and the role of schools in supporting SRH for learners during the pandemic. Learners and stakeholders reported that the SRH of young people was affected by alcohol misuse, poor SRH knowledge and few pathways to link learners with services. Stakeholders working with schools reported that a lack of access to biomedical interventions (e.g., contraception) increased learner pregnancies. Gender-based violence in learners’ households was reported to have increased during the COVID-19 pandemic related to loss of income. School closures disrupted the provision of a safe space to provide SRH and HIV-education through Life Orientation lessons and school nurse talks. This loss of a safe space also left learners vulnerable to sexual and physical violence. However, once schools re-opened, daily COVID-19 screening in schools provided the opportunity to identify and support vulnerable children who had other social needs (food and uniforms).

**Conclusion:**

The COVID-19 pandemic may have increased SRH needs and vulnerability of school-going children in a high HIV-burden rural setting. School shutdowns reduced the opportunity for schools to provide a vital safe space and information to enhance SRH for adolescents. Schools play a vital health promotion and social protection role.

## Introduction

Adolescents in South Africa face the interlinked epidemics of poor sexual and reproductive health (SRH) and high HIV-incidence in a context of exceedingly high youth unemployment, orphanhood, and violence (Dellar et al., 2015; Human Sciences Research Council (HSRC), 2017; Francis et al., 2018). Despite advances in biomedical HIV prevention tools that can reduce HIV transmission and acquisition to zero, two out of every seven new HIV infections globally in 2019 were among young people (15–24 years) with adolescents and young women being at greatest risk of HIV acquisition (UNAIDS, 2021).

Early, unintended pregnancies among schoolgirls exposes them to socioeconomic challenges, sexually transmitted infections (STIs) and HIV, and the discontinuation of their education during that time exacerbates the situation by reducing their future economic and job opportunities. Teenage pregnancy is a major cause of school dropouts and is associated with higher poverty, poor performance and grade repetition in South Africa (Jochim et al., 2021). In South Africa in 2020, teenage pregnancy statistics were estimated as 33 899 babies born to mothers who were 17 years old or younger, with 499 children aged 10 to 13 years giving birth (Statistics South Africa, 2021). Between 2018 and 2019 there was a surge in adolescent pregnancies in various parts of South Africa, and more recently during the COVID-19 pandemic (Jonas, 2021). This suggests challenges in access to and uptake of reproductive health among female learners.

In uMkhanyakude district in rural KwaZulu-Natal, South Africa (SA), although HIV incidence is dropping among young people, it remains high with an HIV-incidence of 4.5 cases per 100 person-years in 2011 to 2015 compared with 2.8 per 100 person-years in 2016 to 2018 among 15–19-year-olds and 7.1 per 100 person-years as compared with 5.8% among 20–24-year-olds, despite widespread antiretroviral therapy (ART) scale-up (Chimbindi et al., 2018; Birdthistle et al., 2021). The setting has a high incidence of teenage pregnancy at 6% per annum among 15–19-year-olds, and one in five 15–24-year-olds has a treatable STI, with the herpes simplex virus (HSV2) incidence at 16.3% per annum (Francis et al., 2018; Shahmanesh et al., 2019). There is a high unmet sexual health need among adolescents. In 2015, only 15% of adolescent girls and young women aged 15–19 years were using contraception while < 50% reported condom use at last sex, and only 32% had tested for HIV in the past 12-months (Chimbindi et al. 2018). There is poor awareness and uptake of effective new HIV-prevention technologies such as HIV pre-exposure prophylaxis (PrEP) in this setting (Chimbindi et al. 2021).

The World Health Organization (WHO) Global School Health Initiative recognises schools as a convenient setting to deliver effective health interventions that promote the well-being of learners to large numbers of young people within a sustainable infrastructure (WHO, 2021). In SA, more than 90% of school-going age (7–19 years) children are in school (Hall, 2015) and school attendance has consistently been associated with lower HIV risk and better sexual health outcomes (Pettifor et al., 2008).

South Africa has the highest number of COVID-19 cases in Africa (34%), accounting for 43% of all reported COVID-19-related deaths on the continent (Abdool Karim & Baxter, 2022). The COVID-19 pandemic has not only directly caused high morbidity and mortality but also exposed the inadequacy of investments in education and public health. The pandemic has shown the persistence of profound economic and social inequalities and disrupted essential systems for health, including SRH and HIV prevention and care programmes. After being declared a global pandemic by WHO, South Africa instituted a national lockdown at the end of March 2020, which restricted movement and closed most parts of the economy, including schools. Globally, there has been widespread concern around the negative impact of school closures on learners. UNESCO indicated that the COVID-19 impact was particularly severe for the most vulnerable and marginalised children and their families. The resulting disruptions exacerbated already existing disparities within the education system for learners. The rippling after-effects of the pandemic and associated responses have directly led to increased exposure to violence and exploitation, early marriages, sexual exploitation of girls and young women, and teenage pregnancies (UNESCO, 2021). Impaired access to healthcare services had a disproportionate impact on HIV testing and SRH services (Dorward et al., 2021; Abdool Karim & Baxter 2022; Freer & Mudaly, 2022). Adolescents (15–19 years) are particularly affected by the closure of schools, community centres, and clinics where many of them obtain SRH education and services (Abdool Karim & Baxter, 2022). A recent review on the impact of the COVID-19 on adolescents’ SRH in Africa found that the pandemic significantly restricted access to SRH services and led to increases in teenage pregnancies and reports of sexual violence (Groenewald et al., 2022). In this paper we investigate the SRH needs of school-going young people in the context of the COVID-19 pandemic in an area of rural KwaZulu-Natal, South Africa with a high burden of HIV.

## Methods

### Study setting

This study is situated in uMkhanyakude district, KwaZulu-Natal, South Africa. The district is predominantly rural with high levels of youth unemployment, significant HIV-burden, mental ill-health and teenage pregnancy among adolescents (Gareta et al., 2021). Health care is mainly nurse-led and provided through the public-sector fixed and mobile clinics with very little or no school-based delivery of health services except for screenings done through the Integrated School Health Teams in primary and secondary schools, including SRH services for older learners (Education 2019).

The HIV and AIDS Life Skills Education Programme Life Orientation (LO) is implemented in the public school system with a focus on learners at the primary and secondary school level. The curriculum is delivered by teachers. The main objectives of the life skills programme are to integrate HIV and AIDS and relevant life skills into the school curriculum as a learning area/subject to prevent and mitigate the spread of HIV infection, and to provide care and support for learners that are infected and affected by HIV.

### Study design

At the time of the study, July–November 2020 and August–November 2021, very little was known about the health needs of the adolescents in South Africa during the pandemic (study 1 July–November 2020) or how schools would respond to the COVID-19 pandemic (study 2 August–November 2021). We thus conducted a qualitative methods exploratory study, consulting various stakeholders, including school learners, teachers, community members and key stakeholders to explore these issues.

### Data collection

We purposively selected four schools to represent rural/urban and high schools/schools in our setting. First, we engaged with the school principals and gatekeepers to introduce the study and set up appointments for recruitment.

We conducted 24 in-depth interviews (IDIs) with principals, teachers and school governing board representatives, Department of Education (DoE) key stakeholders and non-governmental organisation (NGO) representatives using semi-structured interview guides. Interviews were conducted telephonically through the Africa Health Research Institute’s (AHRI) call centre unit. All calls were recorded, transcribed and translated.

Focus group discussions (FDGs) were conducted by telephone. Each focus group was arranged by a research assistant who explained the study to prospective participants and provided consent forms and formed a WhatsApp group to decide on the suitable internet-based application to use (WhatsApp, Zoom or Microsoft Teams) for the call. WhatsApp was also used to agree on the date and time for the group discussion and the provision of information on how to join the call.

For the FDGs with young people attending school, the research assistant worked with the Life Orientation teachers to introduce the study and organise interested learners. All learners were above 18 years old. FDGs with community members were conducted with parents/guardians of young people of school-going age residing in our study community. These focus group participants were recruited using a snowballing approach. Peer navigators were approached and invited to participate in FDGs through a similar process as that used to recruit learners. These peer navigators are young people aged 18–30 working at the Africa Health Research Institute (AHRI) in youth-focused HIV prevention programmes.

We conducted eight FGDs with high school learners, peer navigators, non-governmental stakeholders and parents/community members. A total of 69 participants were recruited in this study: 45 participated in FGDs and 24 in IDIs. Table 1 shows the participants recruited per section of the study.

All interviews were conducted in isiZulu, the local language, by two trained native speaker research assistants. Verbal consent was obtained telephonically and recorded. All the interviews and consenting processes were audio recorded, transcribed verbatim and translated into English and analysed iteratively for themes. Interviews and discussions lasted between 30 and 60 minutes. The main challenge was with network connections, resulting in some interviews or discussions being shorter than others or having fewer participants per group.

Weekly debriefing sessions were conducted with the first author (NC) and the research assistants who reviewed interview summaries and notes to capture and reflect on the interview context and identify emerging codes and themes. We used a thematic analysis approach (Braun & Clarke, 2006) and Nvivo 12 (QSR International) software to manage the data for analysis. Emerging themes in the data were identified and coded using the Nvivo software, which was then used to help us identify recurring patterns. Themes were determined by the study aims which had informed the content of the interview guides as well as inductive development of codes as they emerged from the data. Two members of the study (NC and UN) read all transcripts to familiarise themselves with the contents and contribute to the identification of the initial codes. NC discussed codes with mentors and supervisors of the study (JS and MS) to group codes with similar or close meanings to generate broader themes. Four main themes related to the COVID-19 pandemic emerged from the data: the SRH of learners in the lead-up to the pandemic; the impact of COVID-19 on learners’ SRH and well-being; the opportunities schools provided to support sexual well-being of learners during the pandemic; and the role of schools in supporting SRH for learners during the pandemic.

### Ethics

The study was approved by the Biomedical Research Ethics Committee, University of KwaZulu-Natal (BREC/00000571/2019). Participation was voluntary and verbal consent was obtained telephonically and recorded prior to data collection.

### Findings

In this study we sought to understand the wider health needs and support available for HIV-prevention for adolescents and young people of school-going age within the school environment, as well as to investigate the effect of the non-pharmacological public health response to COVID-19 on the health and well-being of school-going children. We present the findings that relate to the sexual health needs of learners in the context of the COVID-19 related lockdown and the effect of the non-pharmacological public health response to COVID-19 on the sexual health of school-going children.

We present our study findings under the following four main themes that emerged from the data: the SRH of learners in the lead-up to the pandemic; the impact of COVID-19 on learners’ SRH and well-being; the opportunities schools provided to support sexual well-being of learners during the pandemic; and the role of schools in supporting SRH for learners during the pandemic.

### Theme 1: Sexual reproductive health context before COVID-19

Learners and key education stakeholders reported that even before the pandemic, SRH of young people was generally affected by alcohol and substance misuse and poor sexual reproductive health knowledge. Community members and parents indicated that school-going young people often faced multiple challenges due to peer pressure to fit in with their peers and their living conditions.

Many learners did not live at home; instead they rented rooms in houses used as boarding houses and that is where young people engaged in transactional sex and used drugs and alcohol, we were told:

You find out that a school kid has 5, 6 boyfriends. For a boy…they are busy over here smoking marijuana, drinking […] They smoke eh [including girls], they are courting here [in the rented places], they have (sex), and some end up getting pregnant. They do not use condoms; you can even tell that

(peer navigator, FGD).

Key stakeholders working with schools and teachers reported that lack of access to and provision of biomedical interventions (e.g., contraception) and HIV services within schools increased learner pregnancies and poor HIV testing uptake. Teachers, principals, and social workers indicated that some policies, such as not providing condoms in schools (which is the decision of individual school governing bodies) hampered SRH support.

There is certain policy, the (condoms) must be put at the gate because some people tell you openly…they tell you that no, I have girlfriends and I have sex with them or (I am) a father of a child, or a mother of their child. Probably the condoms should be available to help for the pregnancy to decrease, isn’t it is hard to get those condoms, the clinic is far. They must be there at school or the security gate at least, okay

(NGO life coach IDI).

Young people reported that there was a lack of access to support for pregnant learners, resulting in pregnancy termination and school drop-out. Further, they reported poor SRH knowledge among the learners and lack of clear pathways to link with services.

A girl from this household is pregnant obviously, they will laugh at her and say we saw it coming (laughing) you see there is no support in that regard. And I think that’s where a person ends up doing things that are not good you see…things like pregnancy termination

(learner, FGD).

However, COVID-19 had exacerbated many of the health and welfare challenges faced by school-going children.

COVID-19 came at the wrong time while there are still these social ills [alcohol and drugs]; it is very harmful. Eh, then there is alcohol because, after all, children do not stop drinking; any decision taken by a drunk person will never be the correct one

(key stakeholder, IDI).

### Theme 2: Role of schools

Stakeholders described the key role that schools play in promoting sexual well-being and mitigating some of the SRH vulnerabilities (through enrichment and providing a safe space) that learners face in their community. They described how this key protective role was lost during the school closures.

#### Health promotion – SRH promotion

Prior to COVID-19, the schools in our study setting relied mainly on Life Orientation lessons, NGOs and Department of Health (DoH) nurses for educating learners about SRH through workshops and health education talks. School health nurses screened and taught learners about SRH and encouraged them to access and take up care using adolescent friendly services at the primary healthcare clinics, such as the ‘happy hour’ where they do not queue or wait for long.

The (nurses) offered to talk to the children […] they just come to the clinic at a certain time. They gave them the time 4 ’clock onwards, they would attend to them without any questions asked

(high school principal, IDI).

Social workers were reported to visit some schools and educate young female learners about teenage pregnancy and conduct anti-substance campaigns targeting male learners. However, these visits were insufficient, even prior to COVID-19, as they were provided only once every three months, due to a lack of human resources and transport to cover all the schools adequately. Health talks also included information on STIs:

That you must be responsible now that you have started having sex, which means you must know that you have to protect yourself. I talked about how dangerous STIs are in their lives, how much damage they can cause to their bodies…I ask, okay, okay, fine, how many sex partners they have. Okay, fine, I also, talked about having (sex) without using a condom; if you know that you have sex with different partners, unprotected it will make you get an STI

(NGO life coach, FGD).

Schools were seen to be safe spaces for providing HIV education and encouraging uptake and retention in services because young people spent most of their time at school and it is easier to learn about diseases, symptoms, prevention and importance of adherence to treatment while still at school. Suggestions were also made for the need to raise awareness and knowledge of newer technologies such as pre-exposure prophylaxis (PrEP) for HIV prevention in learners as young as those in primary schools to prevent them from getting infected.

So, if you are infected with HIV because we don’t use condoms…that is why I am saying these Grade 6, Grade 7 children need the information about sex. If they become sexually active it is important for them to be protected with a condom, they must take PrEP so they decrease the chance of getting HIV because they like older people

(peer navigator, FGD).

All of these wider sexual health promotion activities were put on hold during the lockdown months (March–May 2020 and again in July–August 2020).

#### Social enrichment – Keeping adolescents safe and occupied

Schools provide sporting and extracurricular activities which are used to teach and engage learners in activities other than academic subjects. These activities were key platforms for integrating and delivering SRH education which were lost during lockdown.

Some local NGOs did provide HIV testing in a few schools which was beneficial, but it was reported that their coverage was limited and did not reach most school children.

Eh, at school there used to be [X NGO] that came and taught us about sports, exercise, (and) just eating a balanced diet. If we wish to test for HIV we can go to the existing containers [temporary shelter] to test at that time

(peer navigator, FGD).

Discussions with peer navigators during the early days of COVID-19 lockdown indicated that young people no longer had access to sports and extracurricular activities. These activities were good for young people in helping them to abstain from and reduce their engagement in risky behaviours.

Eh…what I can say is that children should get something that will keep them busy so that they will be able to abstain…What is important is that a child must get something to keep him/her busy like being involved in sports, there are lot of things they can do, others are attending churches, others are singing and dancing, there are different things that children can do which can help them

(peer navigator, FGD).

#### Schools as a social safety netand how it relates to SRH

Schools play a key social role in supporting menstrual hygiene in our setting; some schools provide sanitary pads donated through NGOs for female learners. This was welcomed by most learners and found to be beneficial as learners did not have to miss school for lack of sanitary wear. However, during lockdown and school closures the provision of sanitary wear for girls ceased.

But now in addition to [participant 4’s name], young people are really privileged now because in schools they get sanitary towels which was not possible (back then). If a person went for her period, she wouldn’t go to school (as) there was no money [to buy proper sanitary wear], or she would use the wrong things that could end up harming her

(peer navigators, FGD).

Food insecurity was another issue described by informants to be a concern in our rural setting due to the high unemployment rates and it often predisposes young people to transactional sex. The Department of Education helps to tackle this through a school feeding scheme. Schools prepare and serve food for both primary and secondary school learners in quintile 1 to 3 (these are no-fee schools, and they are characterised by the poverty level of the community around the school, as well as certain infrastructural factors) to help supplement their diet. One teacher indicated that malnutrition was prevalent among learners, and children who were living alone depended on school meals and received an extra helping to take home. The school feeding programmes were disrupted during the initial COVID-19 hard lockdown and this increased learner vulnerability.

A lot of learners were dependent on the meals that were provided at the school. This also affected parents so much, the lockdown brought a lot of suffering to many households. Sometimes you will find that there is an 18-year-old who takes care of younger siblings, and all these kids depend on the school feeding scheme

(NGO, life coach IDI).

### Theme 3: Adversity during lockdown

#### Sexual reproductive health challenges and abuse

Respondents reported increases in rape cases and sexual and physical abuse of learners during the lockdown. Loss of jobs, unemployment and poverty due to lockdown restrictions exposed young girls to increased risks. Incidents were reported of relatives taking advantage of young girls due to increased contact time, especially when young people were not supervised.

And to make matters worse, you will find that an uncle will lose his job due to COVID-19 and will spend his whole time plotting to take advantage of his niece and as a result the young girl becomes a victim of rape

(NGO, life coach IDI).

Teachers reported that COVID-19 resulted in increased teenage pregnancies as the children spent less time in school — due to rotational learning implemented by the Department of Education to increase social distancing in schools and lack of adult supervision at home indicating the important protective role of keeping children in school.

Those children had a disturbance and because they don’t go to school often, they sometimes meet and play together then they might end up doing naughty things because they are not going to school anymore. And parents are working they are not always with them all the time that’s how kids end up making each other pregnant because they don’t always have adult supervision

(community member, parent IDI).

Teenage pregnancy was reported to be a huge issue in the area and the main reason for school dropouts among the young girls.

#### Mental and psychosocial challenges

One of the key themes that emerged was the significant toll that the pandemic placed on the mental health of learners, and how this in turn directly or indirectly impacted on SRH. Substance abuse is known to have negative effects on young people’s SRH outcomes and is associated with less likelihood to report condom use resulting in STIs, unplanned pregnancies and partner violence. Alcohol and substance abuse were reported to have increased during lockdown in our study. Alcohol was easily available, even during times when the sale of alcohol was banned because people brew their own beer in their homes. There was also reportedly increased smoking of marijuana, particularly by young girls, as a coping mechanism for the stress of the COVID-19 impact. This increased their risk of experiencing violence as they would often sneak out at night to smoke.

During the lockdown there were a lot of reported cases which included rape, substance abuse, and gender-based violence. Sometimes the learners would report that their mothers were experiencing gender-based violence. Substance abuse was very rife during the lockdown. People were under a lot of stress, so they turned to drugs for comfort and because this is a rural area alcohol is easily available and even if it is banned from the shops, people brew their own. Another issue we are faced with is currently is “insangu” [marijuana]

(NGO life coach, IDI).

One community member explained how loss of income due to COVID-19 impacted on the households and the lives of learners. Having to share the same living space for longer periods without income resulted in parents fighting in the presence of their children as they were not in school.

Parents lost their jobs, and they will be stressed […] And most of the time when parents are together for a very long period and not working, they fight all the time, they will start saying that we are hungry in this house and your father is failing to provide

(community member, parent IDI).

Extended periods of separation of learners from one another caused fear and anxiety among young people as they feared this was the end of the world. Learners spent a lot of time on social media:

They began to spend more focus on their phones, watching videos of how people who died from COVID-19 were buried and all that really posed a great stress and anxiety on their well-being

(high school teacher, IDI).

It was realised there was a need to support learners as they went through the trauma of COVID-19 and encourage them to learn to live with the virus but also acknowledge the impact of COVID-19 related deaths:

But while we are saying that, we need to also put in mind that people are dying in front of these children some of them are even orphans due to COVID-19, so we need to also take that into consideration

(community member, parent IDI).

Some learners who were staying far from their homes had to return home or transfer to other schools which caused emotional and financial strain on the parents and the learners.

My child was studying at [area’s name] then they heard that they are dying at (area’s name) then they cried and wanted to come back, can you imagine the money paid for the whole year in a private school and now the child had to come back to study here and start over? I was not going to leave my child while saying, ‘Mom, COVID-19 is high at [area’s name], I am scared’ you can see that it harmed them; we also lost jobs

(community member, parent, IDI).

Parents and learners were saddened when, because of the disruption of the COVID-19 pandemic to schooling, their children failed a year and could not be promoted to the next grade. An NGO life coach explained:

It has affected them negatively. One, they are always under pressure of not knowing if they will pass or fail. Two, they are always anxious if the schools will be closed, and their syllabus will be affected. Three, they also experience loneliness because they do not see their friends as often as before, when they do see each other, they must maintain social distance, they cannot hug, touch one another, or interact just like how they used to before all this new normal has a huge negative impact on the learner’s mental well-being. We are the support system for learners at school, they come to us for consultations, where they express their feelings and share their fears of COVID-19. During the consultations learners will share that they have panic attacks or assume they have developed one or two COVID-19 symptoms. So as much as learners are emotionally affected, they are far better off than their teachers because they have us and we are always available to counsel them when they require help and should the matter be beyond us, we do refer them for professional assistance.

The government introduced a social grant for the unemployed youth during the lockdown to provide relief and support for individuals and household, but this grant caused several problems:

I am referring to the R350 unemployment grant. […] a lot of students would be absent from school and some they need to queue the day before to get assistance. Have you ever seen how long those queues get? Also, the government allocated this money to assist the unemployed youth so that they can be able to have some sort of income, indirectly the grant caused more damage than good. Some of them will unintentionally fall into these social ills, like for instance they will buy alcohol with it because they are already in town and have cash on hand

(NGO, life coach, IDI).

### Theme 4: Opportunities – COVID-19 health promotion and screening vulnerabilities

Despite the many challenges, schools continued to provide social support and health promotion throughout the lockdowns. There was overwhelming support for the school feeding schemes that continued during lockdown which illustrated the social role that schools provide.

Schools were seen as places young people could learn about COVID-19, be monitored and ensure they adhere to COVID-19 prevention strategies, unlike at home. One stakeholder indicated that HIV was no longer ‘killing’ people because of high awareness of prevention and treatment and care support, which was still lacking from COVID-19.

Ah, for me I think the school is a safe place to be than other places, you see when they are at school, even though we always remind them that masks need to be on their mouths and nose all the time so I think if they are not at school they do as they please, there is no discipline and there is no one who cares enough to ensure that their lives are safe and always remind them that they will get sick if they don’t wear a mask

(high school teacher, IDI).

When schools restarted, the daily screening for COVID-19 symptoms conducted each morning as learners arrived at school provided space to identify and support vulnerable children who had other social needs such as food and uniforms. One principal indicated,

When you record in the morning that they are there, then you see the way they are dressed, you can see that no, this child needs an intervention, maybe the jersey is no longer right. In the morning, it’s one-on-one, you take their temperature looking at them until you finish them all, seeing the children that no, [colleagues] please come closer to this child, I [noticed] that they are not well. So, we prepare for that person [to be referred and helped].

## Discussion

Our findings show that the COVID-19 pandemic exacerbated the existing SRH vulnerability and risk of school-going young people, whilst simultaneously removing the protective role that schools provide, namely sexual health promotion, enrichment, and provision of safe spaces. COVID-19 pandemic prevention measures, such as hard lockdowns which limited access to services, restricted young people’s access to life-saving SRH services including contraception, HIV prevention such as condoms, PrEP and STI treatment. This has a huge impact in our setting where pre-COVID uptake of SRH and HIV prevention services among young people was already poor (Baisley et al., 2018; Francis et al., 2018; Gourlay et al., 2019).

Teenagers from low- and middle-income countries are at risk of poor SRH, which can lead to unwanted pregnancies, unsafe abortions, sexual assault, and sexually transmitted infections (UNAIDS. 2021). Prior to the COVID-19 crisis, young people in our setting experienced substantial challenges with access to critical SRH information and services such as HIV testing, condoms and contraception and this improved with the roll-out of combination HIV-prevention programmes targeted at young people which were implemented between 2016 and 2018 (Zuma et al., 2020). We found increased teenage pregnancy being reported to have occurred during the lockdown period. This finding indicates the effect of the COVID-19 restrictions on learners’ access to SRH services and support and their ability to identify pathways to link into care during the lockdown period as was found elsewhere in South Africa (Jonas 2021). Schools provide consistent and accurate SRH information through the curriculum-based Life Orientation subject and peer support through peer buddies in some schools. The Integrated School Health programme provides screening and vaccinations for the lower grades and SRH education and support for referral for the upper grades (Education 2019). In the face of the COVID-19 pandemic these services were disrupted, and this may have contributed to the increased teenage pregnancy observed. Indeed, increased teenage pregnancy was also observed during the lockdown period in most parts of South Africa as well as in other African countries such as Kenya, Zimbabwe and Uganda (Schwikowski 2021; Murewanhema et al., 2022). It is vital that governments ensure that restrictions on movement do not limit access to SRH information and services and they should prioritise and fully fund SRR as part of their COVID-19 response plans.

Disrupted learning, home schooling, rotational learning and boredom were reported to have created an environment where young people had space and time to engage in risky behaviours, including unprotected sex, particularly those living alone without parental/adult supervision. In addition, easy access to alcohol (despite sales of this being banned) and addictive substances, including marijuana, among this age group during lockdown worsened the situation. Most pregnant teenage girls often do not return to school and are more likely to end up in violent relationships or informal employment and sex work (Nordhues et al., 2021, Murewanhema et al., 2022).

Women and girls are more vulnerable to domestic, physical or sexual assault by an intimate partner during global health crises and such assaults are often linked to alcohol and substance abuse (Nordhues et al., 2021). Similarly, in our study, an increase in rape cases, sexual abuse and violence among girls and women during the COVID-19 pandemic lockdown was reported. Women experiencing GBV are at increased risk of contracting HIV and are less likely to report the case or seek medical or legal help (Kumar et al., 2020). Hard lockdown restrictions impacted negatively on the mental health and well-being (Nordhues et al., 2021) of learners, increasing anxiety and fear among them, similar to what has been found in other settings (Elharake et al., 2022). Understanding the mental health effects of the COVID-19 pandemic on children is important for creating timely interventions that will improve young people’s mental well-being, especially in a setting where common mental disorders among young people is already high (Mthiyane et al., 2021) and in a context of high alcohol and drug abuse among young people (Zuma et al., 2020).

Staying in education has been shown to protect girls from acquiring HIV (Pettifor et al., 2008). Our findings show that schools are not merely places of learning but play critical social roles such providing meals for children and families as well as safe spaces for learning about SRH and provision of sanitary wear for girls.

Our study adds to the growing body of knowledge that suggests schools are an important setting to deliver effective health interventions that promote the well-being and improved sexual health outcomes for learners (Pettifor et al., 2008; WHO, 2021). Much work has been done on the mental health impact of the COVID-19 pandemic and associated non-pharmacological control measures on young people but these are mainly in high-income settings (Chadi et al., 2022). In this paper, we have shown some of the negative impacts on SRH and the mental health of learners in the short term in a poor rural setting. Further work needs to be done to monitor the medium-term effect, e.g., on HIV incidence, STIs and teenage pregnancy and mitigate against the longer-term effects of the COVID-19 pandemic on young people.

## Conclusion

We found schools in rural KwaZulu-Natal are perceived as a safe space to reinforce and deliver interventions and promote SRH and health and well-being during pandemics. However, the COVID-19 pandemic may have increased SRH needs and vulnerability of school-going children in our high HIV-burden rural setting. School shutdowns reduced the opportunity for schools to provide a vital safe space and information to enhance SRH for adolescents. There is need to strengthen and protect the health promotion and social protection role that schools provide, especially in the context of HIV and sexual health during pandemics by ensuring that non-pharmacological public health responses do not impact negatively on school-going children’s ability to access SRH information and services.

## Figures and Tables

**Table 1.** Participants enrolled in the two studies

Participants	Study 1 July–November 2020	Study 2 August–November 2021	Total
In-depth interviews			
Principal and deputy principal	2 females; 2 males		4
Life orientation teacher	2 females; 2 males	3 females	7
Educational social worker	1 female		1
School governing board member	2 females; 1 male		3
Non-governmental organisation stakeholder life coach	2 females	1 female	3
Department of Education stakeholder inspector		1 male	1
Community member/parents and guardians		3 females; 2 males	5
Focus group discussions			
Community member/parents and guardians	*Two focus group discussions* 9 females; 7 males		16
Learners	*Four focus group discussions* 7 females* were enrolled but only 1 remained to the end of the call; 7 males* were enrolled but only 3 remained to the end of the call; 9 females; 4 males		17
Non-governmental organisation stakeholder life coach	*One focus group discussion* 3 females* were enrolled but only 2 remained to the end of the call		2
Peer navigators	*One focus group discussion* 6 females and 4 males (mixed group)		10
Total			69

* Participants dropped from the call due to poor network connections
